# Residential Mercury Spills from Gas Regulators

**DOI:** 10.1289/ehp.8401

**Published:** 2006-02-27

**Authors:** Daniel Hryhorczuk, Victoria Persky, Julie Piorkowski, Jennifer Davis, C. Michael Moomey, Anne Krantz, Ken D. Runkle, Tiffanie Saxer, Thomas Baughman, Ken McCann

**Affiliations:** 1 Great Lakes Center for Children’s Environmental Health, John H. Stroger Jr. Hospital of Cook County, Chicago, Illinois, USA; 2 University of Illinois–Chicago School of Public Health, Chicago, Illinois, USA; 3 Toxikon Consortium, Chicago, Illinois, USA; 4 Division of Environmental Health, Illinois Department of Public Health, Springfield, Illinois, USA

**Keywords:** elemental mercury, environmental, gas regulator, public health, residential, screening, surveillance

## Abstract

Many older homes are equipped with mercury-containing gas regulators that reduce the pressure of natural gas in the mains to the low pressure used in home gas piping. Removal of these regulators can result in elemental mercury spills inside the home. In the summer of 2000, mercury spills were discovered in the basements of several Chicago-area homes after removal of gas regulators by gas company contractors. Subsequent inspections of approximately 361,000 homes by two northern Illinois gas companies showed that 1,363 homes had residential mercury contamination. Urine mercury screening was offered to concerned residents, and results of urine bioassays and indoor mercury air measurements were available for 171 homes. Six of these 171 homes (3.5%) had a cumulative total of nine residents with a urine mercury ≥ 10 μg/L. The highest urine mercury concentration observed in a resident was 26 μg/L. Positive bioassays were most strongly associated with mercury air concentrations > 10 μg/m^3^ on the first floor [odds ratio (OR) = 21.4; 95% confidence interval (CI), 3.6–125.9] rather than in the basement (OR = 3.0; 95% CI, 0.3–26), and first-floor air samples were more predictive of positive bioassays than were basement samples. Overall, the risk of residential mercury contamination after gas regulator removal ranged from 0.9/1,000 to 4.3/1,000 homes, depending on the gas company, although the risk was considerably higher (20 of 120 homes, 16.7%) for one of the contractors performing removal work for one of the gas companies. Gas companies, their contractors, and residents should be aware of these risks and should take appropriate actions to prevent these spills from occurring and remediate them if they occur.

Elemental mercury is a silvery metal that is liquid at room temperature. It has a vapor pressure of 0.00185 mm at 25°C, and sufficient amounts can move from the liquid phase into the vapor phase to exceed permissible limits for inhalation exposure. Human absorption of elemental mercury occurs primarily through inhalation of mercury vapor. Mercury has been used in many common household products such as glass thermometers, barometers, thermostats, and fluorescent lights. Exposure to mercury vapor occurs when these sealed products are broken and the mercury contained inside is accidentally released. Public health surveillance of mercury spills in 14 states during the time period 1993–1998 showed that 16.7% of reported spills occurred in private residences, second only to health care facilities (Zeitz et al. 2002). Serious outbreaks of mercury poisoning have occurred, primarily in children, when large amounts of metallic mercury have been unwittingly brought into the home for play [Centers for Disease Control and Prevention (CDC) 1991, 1995; Cherry et al. 2002; Fuortes et al. 1995; Tominack et al. 2002). The Agency for Toxic Substances and Disease Registry (ATSDR) has set a minimum risk level for chronic inhalation exposure of 0.2 μg/m^3^ (ATSDR 1999). Carpi and Chen (2001) believe that up to 10% of households may have levels of airborne mercury > 0.3 μg/m^3^ caused by historic accidents with mercury-containing devices.

Before 1961, many homes in northern Illinois were equipped with gas meters connected to mercury-containing gas regulators. On average, these regulators contained about 136 g (2 teaspoons) of elemental mercury in a small cup. The purpose of the regulator was to reduce the pressure of the natural gas in the mains to the low pressure used in home gas piping. The mercury acted as a seal to the relief vent in the event of a pressure surge. As technology progressed, newer gas regulators were developed that did not use mercury. A diagram and photograph of a mercury-containing gas regulator are shown in Figures 1 and 2.

Beginning in the 1960s, gas companies in northern Illinois began moving gas regulators from inside the home to outside the home. The removal process involved careful removal of the mercury from the cup in the regulator and transfer to a larger container before the regulator was removed from the home. An overspill container was used during removal of the mercury to prevent it from spilling onto the floor.

On 22 July 2000, a resident of a Chicago suburb called the Illinois Poison Center after he discovered elemental mercury on his basement floor beneath an area where a gas regulator had been recently removed by a contractor for the gas company. The Poison Center referred the case to the Illinois Department of Public Health (IDPH) and the ATSDR for investigation. The IDPH contacted the gas company and learned that it was investigating three other spills in neighboring homes. The IDPH and the ATSDR also contacted the U.S. Environmental Protection Agency (EPA). In this article we present one of the index case families, describe the public health response, present the results of environmental and health surveillance in affected homes, and share the lessons learned from this investigation.

## Case Report

On 12 June 2000, a contractor for a northern Illinois gas company removed a gas meter and regulator from the basement of a suburban Chicago home and replaced it with a new meter and regulator located on the outside of the building. The family who lived in the home included a 35-year-old male, his 38-year-old wife, and their 9-year-old son. The family kept cats as pets. The gas meter and regulator had been located in a windowless work room in the basement. A gas furnace was located in a separate area of the basement, distant from the work room. A part of the basement had been finished and converted into a carpeted video room that was separated from the work room by two partially finished walls. The work room had been vacuumed once in the weeks after 12 June. On 27 July, the father discovered 1–2 teaspoonfuls of elemental mercury on the floor of the work room, beneath the area where the gas meter and regulator had been removed. The cats’ litter box was located in this same area. In June and July, the father had spent approximately 8 hr per week in the work room, and his son had spent several hours per day watching television in the video room. The mother spent the least amount of time in the basement.

A screening environmental investigation on 27 July with a Jerome mercury vapor analyzer (MVA) measured 27 μg/m^3^ at the front door, 54 μg/m^3^ at the kitchen entrance, and 78 μg/m^3^ on the staircase leading to the basement. The family was instructed to ventilate the house, and a follow-up environmental investigation on 31 July showed breathing zone air mercury concentrations of 3.0 μg/m^3^ in the living room and dining room, 4.1 μg/m^3^ in the hallway, and 3.2–6 μg/m^3^ in the bedrooms. The marked drop in air mercury concentrations was most likely due to the ventilation, although other possibilities include measurement error during the initial sampling due to instrument interference (e.g., cat urine) or differences in measurement heights between the two sampling dates.

The family was relocated to a hotel while the spill was cleaned up and contaminated areas of the home remediated. The son, who had a history of frontal headaches, complained of worsening of his headaches over the previous several weeks. He had developed a facial rash that was treated by a dermatologist with a steroid cream. The father complained of new-onset fatigue. The mother was asymptomatic. No family members exhibited erethism, gingivitis, tremor, or acrodynia. On 29 July, the son had a blood mercury level of 16 μg/L (laboratory reference range ≤ 13 μg/L) and a 24-hr urine mercury level of 25 μg/L. The father had a blood mercury level of 11 μg/L and a 24-hr urine level of 23 μg/L. The mother’s blood and urine mercury levels were < 10 μg/L. After removal from exposure, rechecks of the child’s 24-hr urine mercury levels were 10 μg/L on 21 August and < 10 μg/L on 13 November. Postremediation sampling of air in the home on 22 August confirmed levels to be < 1 μg/m^3^.

## Public Health Response

As more information became available and the scope of the problem became apparent, the office of the Illinois Attorney General (IAG) was contacted and began to oversee response efforts. The IAG requested that the U.S. EPA develop formal remediation and clearance sampling protocols to be used throughout the response. The IAG also organized a multiagency task force to organize and coordinate response efforts. The task force consisted of representatives from the IDPH, the ATSDR, the U.S. EPA, the IAG, the Cook County Health Department, the Chicago Department of Environment, the Illinois Commerce Commission, the Illinois Poison Center, and the Illinois Environmental Protection Agency (IEPA). With oversight provided by the IAG, the purpose of the task force was to provide instruction and oversight to gas companies and their contractors; establish guidance on mercury vapor action and clearance levels; define cleanup objectives; develop clearance sampling methods; and provide information to concerned residents.

The gas company entered into consent agreement with the IAG that required compliance with protocols and procedures adopted by the task force and oversight of all activities by the IAG. On 31 July 2000, the IAG’s office informed the gas company that further investigation and cleanup of potentially contaminated homes were required. On 3 August 2000, the gas company compiled a list of about 85 homes where the contractor had performed a mercury regulator removal within the last year. This expanded the affected area to nine other suburban communities. The company later determined that the actual number of homes where the contractor might have worked within the last year was closer to 120. Inspections were conducted in these homes, and 20 (16.7%) were found to be contaminated and required cleanup.

The gas company established a hotline to identify homes where other subcontractors may have recently removed a regulator. On 25 August 2000, the company tested a home where one of its own technicians had removed a mercury regulator in 1989. Elemental mercury was found in the basement near the location of the former meter. The company then decided to screen all homes where either subcontractors or its employees may have removed a mercury regulator in the past. On 26 August 2000, the company announced that > 200,000 homes would be inspected and screened. In September 2000, a second gas company servicing northern Illinois discovered mercury contamination in one of its customer’s homes. This second company projected testing 90,000 homes in two separate northern Illinois service areas. This company also entered into a voluntary agreement with the IAG and agreed to comply with all task force recommendations. Along with customer homes, the gas companies identified scrap yards and service centers that were potentially affected. Monitoring at these locations was overseen by the IEPA and is not presented here.

The task force worked with the company to develop a plan to inspect and screen homes efficiently and effectively. The task force agreed that those homes from which a mercury regulator was most recently removed or homes with young children or pregnant women would be screened first. Homes with visible mercury present were given top priority. Because adequate records were not available to identify the homes that had regulators removed, all potential homes were screened. Screening included a visual inspection to determine if a meter and regulator had been removed in the past, as well as a follow-up visit with an MVA to monitor mercury vapor concentrations. Visual screening preceded MVA monitoring until enough MVAs became available to allow screening teams to conduct both a visual survey and MVA monitoring in the same trip to the home.

The ATSDR and the IDPH established a cleanup clearance level for mercury concentrations in household air of < 1 μg/m^3^ and a relocation action level of 10 μg/m^3^ in a living area. The gas company offered to relocate residents until the cleanup was complete. As part of the consent agreement between the IAG and the gas companies, the IEPA and IDPH performed quality-control oversight of the companies’ field activities. Mercury air concentrations were rechecked at several hundred residences by the IDPH, U.S. EPA, and contractors of the gas companies and were found to be consistent with gas company results.

The IDPH established a hotline to answer mercury-related questions from residents and health care providers. This hotline received > 4,000 calls from August 2000 to April 2001. In addition, a mercury educational pamphlet and a fact sheet were made available on the IDPH website (IDPH 2006) and to interested persons. The gas companies also developed and implemented public information programs that included websites, pamphlets, letters to affected parties, and reports of findings. The company websites, which were updated on a daily basis, included progress reports, health information, links to health agencies, and diagrams and pictures intended to assist with identifying mercury-containing regulator installations. In addition, each community was informed of the residences affected by this program through a secure web link.

### Environmental surveillance

The Mercury Task Force developed an inspection and sampling protocol for mercury contamination within a residential home that was followed by gas company contractors (ATSDR 2001). A visual inspection was initially conducted at each residence to determine if a mercury regulator had been removed and if visible mercury was present. Before the visual inspection, staff was provided information about mercury, the operation and identification of mercury regulators, the installation process of natural gas equipment, and how to determine the previous inside location of a mercury regulator. The inspection process included an outside inspection of the home, contact with the customer, and, if granted permission by the customer, an inside inspection of the home. The outside inspection included determining if an outside meter, a “pin-off” tee, or a vent pipe was present. (The pin-off tee is the name given to a style of shutoff valve used when residential gas service installations were first being installed outside the home). The installation or existence of pin-off tees was a criterion used for determining whether the home had ever had an inside mercury-containing gas regulator. The locations were noted, and an attempt was then made to contact the customer. Sample dialogue was developed for the initial contact with the customer to better facilitate accurate communication. Inspection personnel provided the customer with information about the inspection process and requested information about the regulator removal procedure and potential mercury contamination in their home. The inside inspection included determining if the home had an inside mercury regulator, the previous location of the mercury regulator, and a careful visual inspection for visible mercury droplets with a flashlight in the suspected location of the previous regulator. Inspectors were instructed to put on new disposable booties and search the area of the old service entrance and location of the regulator by scanning with a flashlight, looking for any small, shimmering puddles or masses. The areas to be inspected included those directly beneath the service entrance and regulator, adjacent surfaces, cracks and crevices in the floor or wall, and joints where the wall meets the floor. A floor plan of the home was developed, and potential areas of concern, including the location of the old service entrance and regulator, were identified and documented for the instrument inspection.

Screening was performed with a Jerome MVA (Arizona Instruments, Tempe, AZ), a Nippon Portable Mercury Survey Meter (Nippon Instruments Corporation, Tokyo, Japan), a VM-3000 Mercury Vapor Monitor (Mercury Instruments, Karlsfeld, Germany), an MVI (Shawcity Limited, Faringdon, Oxon, England), and a Lumex RA-915+ (Ohio Lumex Company, Twinsburg, OH). Confirmatory sampling was conducted after remediation and before reoccupancy of the home following sampling and laboratory analysis methods of either the National Institute for Occupational Safety and Health (NIOSH; Method 6009) (NIOSH 1994) or the Occupational Safety and Health Administration (OSHA; Method ID-140) (OSHA 1991). The ATSDR (2001) developed recommendations for air mercury concentration action levels to guide remediation efforts (Table 1).

Gas company A screened 301,000 homes. Of these, 1,308 were contaminated with mercury (0.43%), and 1,033 of these homes were remediated. Gas company B screened 60,000 homes; 55 of these were found to be contaminated, and all were remediated. The overall risk of finding a contaminated home among those screened was 4.3/1,000 for company A and 0.9/1,000 for company B. The risk was considerably higher (20 of 120 homes, 16.7%) for one of the contractors performing removal work for one of the gas companies.

### Urine mercury screening

Under the consent decree, gas companies offered free urine mercury screening to residents who believed they may have been exposed to mercury after removal of gas regulators from their homes. The urine screening protocol was developed by the IDPH and consisted of completion of an exposure questionnaire and collection of a 24-hr urine sample for mercury analysis. Urine sampling was conducted by local hospitals and clinics, and the samples were submitted to several commercial laboratories that had existing contracts with these health care institutions.

**Figure 3:**
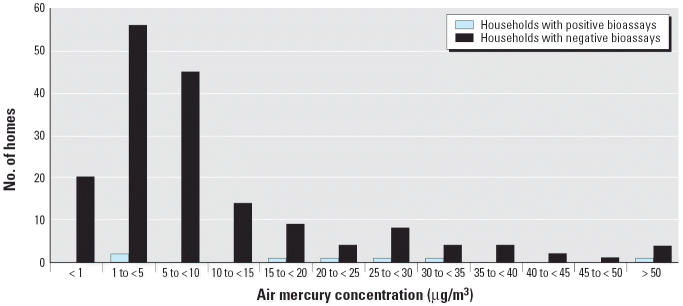
Distributions of mean air mercury concentrations for households with and without positive bioassays. Households with a positive bioassay were those in which ≥ 1 residents had a urine mercury concentration ≥ 10 μg/L.

Urine mercury results for 625 individuals were provided to the IDPH. Of these, 420 were identified as residents of 171 homes for which mercury air monitoring data were also available. A positive mercury bioassay was defined as a 24-hr urine mercury concentration ≥ 10 μg/L. Nine of the 625 residents (1.4% of residents) from 6 of these 171 homes (3.5% of homes) had positive bioassays. These positive bioassays ranged from 10 to 26 μg/L. The frequency distribution of the mean air mercury concentration and urine bioassay results (households in which at least one resident had a urine mercury concentration ≥ 10 μg/L) in these monitored households is presented in Figure 3.

Positive bioassays were more strongly associated with maximum mercury air concentrations > 10 μg/m^3^ on the first floor [odds ratio (OR) = 21.4; 95% confidence interval (CI), 3.6–125.9] than with those in the basement (OR = 3.0; 95% CI, 0.3–26.0). For basement air concentrations, the relative odds of having a resident with a positive bioassay became significant only when the maximum air concentrations exceeded 25 μg/m^3^ (OR = 8.8; 95% CI, 1.0–77.2). The sensitivity, specificity, and predictive values of increasing maximum mercury air concentrations in predicting homes with residents with positive bioassays are presented in Table 2. The predictive value of a negative environmental sample was high for both basement and first-floor samples. In contrast, the predictive value of a positive environmental sample was low for the basement (5–10%) but moderate (22–40%, depending on the cutoff) for first-floor samples.

## Discussion

Removal of mercury-containing gas regulators poses a hazard of residential mercury contamination if the mercury is spilled during the procedure. Factors that may contribute to spills during the removal operation include lack of awareness on the part of the technician of the potential for mercury spillage and necessity for control and mitigation; not noticing small, inadvertent spills; work in difficult field conditions, such as tight quarters, making spill control problematic; and lack of specific spill control practices. It is also possible that some mercury spills occurred during the installation of these gas regulators, although the extent of this risk is unknown. The risk of having current mercury contamination for homes that had gas regulators removed ranged from 0.9/1,000 to 4.3/1,000 depending on the gas company. The risk was as high as 16.7% for one contractor involved in regulator removal. Information on other contractors or gas company employees performing the work was not available. In the subset of 171 homes whose residents submitted urine tests for mercury, 1.4% of residents had urine mercury levels ≥ 10 μg/L. Moreover, the maximum urinary mercury concentration observed was 26 μg/L. We are not aware of any cases of acrodynia or clinically overt mercury poisoning as a consequence of these exposures. However, the screening programs that were implemented by local public health agencies in response to this incident were not designed to look for subclinical effects of mercury exposure.

In addition to the air concentration, factors that affect doses received by individuals include duration of exposure, ventilation rate, and individual toxicokinetics. In our study homes, positive urine mercury in residents was more strongly associated with air mercury concentrations on the first floor than in the basement, even though mean basement concentrations were considerably higher. We suspect this is because residents typically spent less time in the basement.

Although mercury concentrations were typically highest in the area of the basement where the spill had occurred, many homes exhibited unacceptably high air mercury concentrations throughout the house. This may be due to air movement throughout the house, the dispersal of mercury by cleaning (especially vacuuming), and inadvertent tracking of elemental mercury by residents or pets. Alternatively, some of these high levels may have been due to previous spills from other, unrecognized sources. Factors that determine air mercury concentrations include the amount spilled, surface area of the droplets (or droplet diameter), floor temperature where the droplets were spilled, type of surface where the droplets were spilled, presence of dust film on the droplet surface, room temperature, and number of air exchanges per hour. Riley et al. (2001) modeled air mercury concentrations after the hypothetical spill of 9 g elemental mercury in a 40 m^3^ room with an air exchange rate of 0.5 air exchanges/hr and an average droplet diameter of 1 mm and arrived at an estimated air concentration of 7 μg/m^3^. By comparison, gas regulators contain on average 135 g elemental mercury. In the case presented here, the family observed 1–2 teaspoonfuls (67–135 g) of elemental mercury on their basement floor. The resulting air concentrations of 27–78 μg/m^3^ and positive bioassays in two of the three family members demonstrate that significant overexposure can result from these types of spills. Fortunately, rapid identification and remediation of the spill limited the duration of overexposure, and blood and urine bioassays returned to normal with the predicted biologic half-lives of 1–3 weeks for blood and 1–3 months for urine (CDC 2005).

The ATSDR has set a minimal risk level of 0.2 μg/m^3^ for mercury in residential air (ATSDR 1999). This level, which relied on data from occupational studies, used tremor as the most sensitive end point and provides an uncertainty (safety) factor of 30 (ATSDR 1999). Although the ATSDR has not set a formal biologic exposure index for urine mercury, urine mercury concentrations < 10 μg/L are considered normal reference ranges by most laboratories. Based on data collected in the 1999–2002 National Health and Nutrition Examination Surveys, geometric mean urinary mercury levels in the general population (with 95% CIs) are 0.343 μg/L (0.299–0.393) for males and females 1–5 years of age and 0.606 μg/L (0.553–0.665) for females 16–49 years of age (CDC 2005).

The clinical laboratories that performed the urine mercury analyses in our study generally set < 10 μg/L as their reference value, and we used ≥ 10 μg/L as bioassay evidence of airborne overexposure to mercury vapor. Tsuji et al. (2003) evaluated mercury in the urine as an indicator of exposure to low levels of mercury vapor. They showed that a correlation between air and urine mercury does exist at airborne mercury levels < 50 μg/m^3^, but that the correlation is reliable only down to concentrations of about 10 μg/m^3^, because below 10 μg/m^3^, predicted urine mercury levels are within reported background ranges. Given that the predicted ratio of air to urine mercury levels at 50 μg/m^3^ ranges from 1:1 to 1:3, the use of a laboratory reference value of ≥ 10 μg/L can screen for environmental exposures to mercury vapor > 10 μg/m^3^ but may miss exposures between the ATSDR minimal risk level of 0.2 μg/m^3^ (ATSDR 1999) and 10 μg/m^3^. In our sample, two homes with residents with positive bioassays had mean air mercury concentrations between 1 and 5 μg/m^3^. In one of these homes, Jerome MVA readings ranged from 4 to 44 μg/m^3^ 6 inches above the floor and from 14 to 33 μg/m^3^ at waist height in the basement at the former regulator location. Basement ambient readings at waist height ranged from 0 to 6 μg/m^3^, whereas first-floor levels were all < 1 μg/m^3^. In the second home, Jerome MVA readings were 5 μg/m^3^ in the basement and laundry room, but generally below the limit of detection on the first floor.

From a public health standpoint, this experience provided valuable lessons for future public health response. Over the course of 1 month, there was a massive jump in scale in the number of homes requiring exposure assessment, from an estimated 120 homes to > 200,000 homes. The initial group of homes identified for inspection included those homes that were serviced by the contractor who performed the work in the first home reporting mercury contamination. Discovery of contamination in homes where work was performed by other contractors or company personnel resulted in a decision to attempt an inspection of all homes that had a regulator moved and dramatically increased the number of potentially affected homes and scope of work. Additional homes identified by a second gas company that services two areas in the Chicago area also increased the scope of work and management of the project. The limitations to carrying out exposure assessment included too few personnel, insufficient numbers of mercury monitors, and laboratories unable to handle the volume of samples. This required prioritization of homes for screening based on visible contamination with mercury and presence of high-risk populations, such as pregnant women and children.

The response required development of protocols for exposure assessment, environmental monitoring, urine mercury screening, and remediation plans based on action levels. Quality-control plans were developed and implemented to monitor the work of gas company contractors.

Environmental monitoring was conducted using two different types of MVAs: gold film sensor and atomic absorption spectrophometric (AAS) analyzers. In our experience, the gold film sensor analyzer could give false positive readings from ammonia compounds and chlorine bleach. Indoor sources of ammonia compounds include tobacco smoke, window cleaner, and many other household cleaners. The gold film sensor analyzer was also sensitive to temperature changes, which could result in unpredictably different readings. By contrast, we found that the AAS analyzer was not affected by any household chemicals and was, for the most part, unaffected by temperature changes. However, when brought inside after being in temperatures < 15°F, the optics would sometimes fog, resulting in erratic readings until the optics unfogged, which could take up to 20 min. The AAS analyzer is also much more sensitive than the gold film sensor analyzer, and U.S. EPA has found that one AAS analyzer, the Lumex RA915, is comparable in sensitivity and accuracy with Hopcalite tubes. In our opinion, AAS MVAs are preferable to gold sensor MVAs for the monitoring of low-level mercury vapor exposures in residential environments.

Resident concerns included both the risk of health effects and potential loss of property values. Relocation of families from their homes compounded problems of risk perception. This experience illustrated the importance of clear risk communication to hundreds of thousands of members of the general public. Public information programs were implemented by the IDPH and the gas companies. The establishment of a mercury hotline by the IDPH, referral of concerned residents to environmental health specialists, education of health care providers, establishment of websites, and media access to knowledgeable specialists were critical to the success of the risk communication effort.

This experience also demonstrated the importance of cooperation and collaboration among the major partners entrusted with protection of public health and the environment. Under the leadership of the office of the IAG, a multiagency task force developed and implemented the response plan. Nongovernmental stakeholders, such as the Illinois Poison Center, the University of Illinois School of Public Health, and the Pediatric Environment Health Specialty Unit at John H. Stroger Jr. Hospital of Cook County, provided needed environmental health expertise. The cooperation of local gas companies in complying with the recommendations of the task force greatly expedited response efforts.

On 10 October 2001, the IAG issued a consent order with the primary gas company requiring them to implement the approved remediation work plan until all homes identified as having a meter removed had been fully evaluated and remediated as needed. The order required the company to implement specific procedures and protocols during future removals, including monitoring before and after meter removal. If contamination does occur during future removals, the consent order requires the company to notify and remediate. The company is required to report annually to the IAG’s office regarding its activities pertinent to the consent order through 2006.

In summary, we present a previously unrecognized source of residential mercury contamination. Based on the available information during the response, the task force recommended evaluating all homes that had mercury-containing gas regulators removed to determine if mercury had been spilled in the homes. From our observations, for every 1,000 homes that had a gas regulator removed, one to four homes, depending on the gas company, had a mercury spill resulting in unacceptably high levels of mercury vapor in the home. For one of the contractors, the risk was much higher: 16.7% (20 of 120 homes). One percent of residents of affected homes had evidence of mercury absorption in the urinary bioassay. Despite this potential for overexposure, the maximum observed urinary mercury concentration was only 26 μg/L. By comparison, Forman et al. (2000) recommend considering chelation therapy for subclinical elemental mercury poisoning in children at urinary mercury levels ≥ 50 μg/g creatinine. In Illinois, public health surveillance did not identify any cases of acrodynia or clinically overt mercury poisoning (manifested by erethism, tremor, and gingivitis) as a result of these exposures. Potential subtle effects in exposed individuals have not been evaluated. Nevertheless, gas companies and their contractors need to be aware of the potential hazard and take steps to prevent spillage of mercury during removal of gas regulators. Residents should also be aware of the hazard and contact their gas company if they find a mercury spill at the site of removal. Clinicians need to be aware of this potential source of mercury exposure and consider adding questions about recent gas regulator removals when taking an environmental history. Guidelines and protocols developed for this Chicago-area response may be helpful tools for public health officials who may be faced with developing future public health responses to large-scale residential mercury exposures.

## Figures and Tables

**Figure 1:**
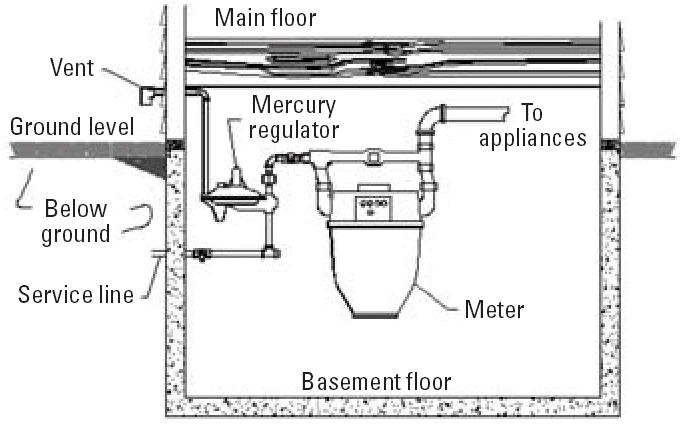
Diagram of a typical inside mercury regulator and meter set.

**Figure 2:**
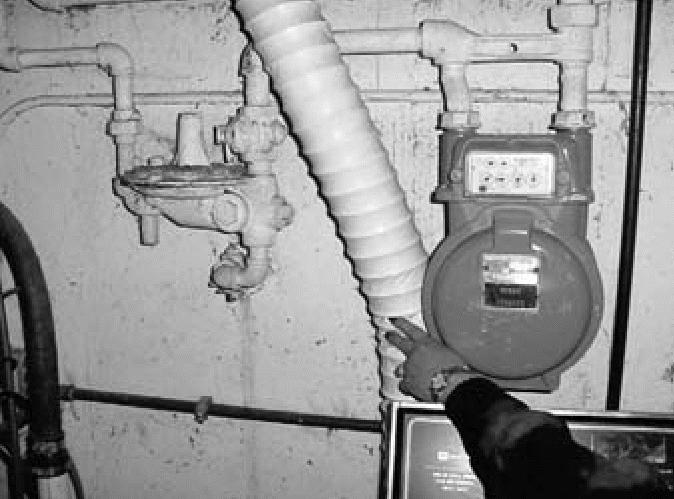
Photograph of a mercury regulator in a basement.

**Table 1:** Recommended action levels by residential air mercury concentrations.

Mercury air concentration (μg/m^3^)	Recommended action
No evidence of spill; no qualitative detection on meter	Level acceptable for occupancy
0.2	Minimal risk level
< 1.0	Level acceptable for occupancy measured by the highest quality data (NIOSH 6009 or equivalent)
≥ 10	Isolate residents from exposure

**Table 2:** Predictive value of maximum air mercury concentrations in identifying households with a resident with urine mercury levels ≥ 10 μg/L.

	Basement				First floor			
Air mercury (μg/m^3^)	Sensitivity	Specificity	PV+	PV−	Sensitivity	Specificity	PV+	PV−
≥ 10	83	37	5	98	50	96	30	98
≥ 15	83	53	6	99	33	96	22	97
≥ 20	83	58	7	99	33	97	29	97
≥ 25	83	64	8	99	33	97	33	97
≥ 30	83	67	8	99	33	98	40	97
≥ 35	83	69	9	99	33	98	40	97
≥ 40	83	73	10	99	33	98	40	97

Abbreviations: +, positive; −, negative; PV, predictive value of maximum air mercury levels in predicting homes with residents with urine mercury values ≥ 10 μg/L.
